# Supplementary material for: Non-viral derivation of induced pluripotent stem cells from the canine umbilical cord
Source: PLoS One. 2025 Jun 18;20(6):e0315161. doi: 10.1371/journal.pone.0315161 (PMC12176200; doi:10.1371/journal.pone.0315161)
Supplement: S1 Table — (PDF) [file pone.0315161.s001.pdf]

List of the primary antibodies.

Antigen	Host	Clone	Conjugate	Source	Cat. No.	Dilution
**OCT4**	Rabbit	Monoclonal EPR17929	Alexa Fluor 488	Abcam	ab283741	1/200
**SOX2**	Rabbit	Monoclonal EPR3131	Alexa Fluor 488	Abcam	ab195358	1/100
**NANOG**	Rabbit	Monoclonal EPR2027(2)	Alexa Fluor 488	Abcam	ab196155	1/100
**SSEA1**	Mouse	Monoclonal MC-480	-	Abcam	ab16285	1/100
**SSEA3**	Rat	Monoclonal MC-631	-	Abcam	ab16286	1/250
**SSEA4**	Mouse	Monoclonal MC-813-70	-	EMD Millipore	MAB4304	1/250
**TRA-1-60**	Mouse	Monoclonal TRA-1-60	-	Abcam	ab16288	1/200
**TRA-1-81**	Mouse	Monoclonal TRA-1-81	-	Abcam	ab16289	1/200
**SOX17**	Goat	Polyclonal	-	R&D Systems	AF1924	1/100
**αSMA**	Mouse	Monoclonal 1A4	-	Agilent Technologies	M0851	1/100
**βIII tubulin**	Mouse	Monoclonal SDL.3D10	-	Merck KGaA	T8660	1/100

List of the secondary antibodies.

Antigen	Host	Clone	Conjugate	Source	Cat. No.	Dilution
**Mouse IgM**	Goat	Polyclonal	Alexa Fluor 488	Thermo Fisher Scientific	A-21042	1/400
**Rat IgM**	Goat	Polyclonal	Alexa Fluor 488	Thermo Fisher Scientific	A-21212	1/400
**Mouse IgG**	Goat	Polyclonal	Alexa Fluor 555	Thermo Fisher Scientific	A-21424	1/400
**Goat IgG**	Donkey	Polyclonal	Alexa Fluor 488	Thermo Fisher Scientific	A-11055	1/400
**Mouse IgG**	Donkey	Polyclonal	Alexa Fluor 488	Thermo Fisher Scientific	A-21202	1/400
