# Supplementary material for: Non-viral derivation of induced pluripotent stem cells from the canine umbilical cord
Source: PLoS One. 2025 Jun 18;20(6):e0315161. doi: 10.1371/journal.pone.0315161 (PMC12176200; doi:10.1371/journal.pone.0315161)
Supplement: Attachment — Submitted filename: response to reviewer.docx [file pone.0315161.s003.docx]

January 27, 2025

To Editor,

Thank you so much for your insightful review of this study. We agree with all of your comments, and the manuscript was thoroughly revised based on your suggestions. The line number in each response was shown as the number in the revised manuscript.

**Reviewer #1**

Thank you very much for your careful review of our manuscript. Our responses to your comments are as follow:

The author described successful IPS-derived from umbilical cord analysis by using non-viral vector, and made it for therapeutic condition for transformation likely by using some transcription factors. Author need to be update protein levels likely proteins levels and cell proliferation rate and, also needs to be included chromosomal abnormality even stemness function checked.
therefore, first of all,

1)immunoblot analysis
2)msc differention cell cycle checkpoint
3)chromosomal abnormality

**Response:**

1)immunoblot analysis

As you suggested, immunoblot analysis may be suitable for measuring protein levels of canine umbilical cord-derived iPSCs. However, the expressions of canine endogenous mRNAs OCT4, SOX2, and NANOG were confirmed by RT-qPCR, and the expressions of stem cell marker proteins were confirmed by fluorescence immunostaining in this study. Therefore, the immunoblot analysis is a future work.

2)msc differention cell cycle checkpoint

Further study is needed to reveal MSC differentiation and cell cycle checkpoint. In addition, these data are being obtained, and the results of additional studies will be published in a new paper.

3)chromosomal abnormality

As you indicated, karyotyping is essential for the clinical application of canine iPSCs. Following this study, experiments were conducted to confirm the reproducibility of iPSC generation. Then, karyotyping of canine umbilical cord-derived iPSCs from other individuals revealed that they contained cell lines with normal chromosome numbers. However, the cell lines in the present study have not undergone karyotyping analysis, which is a topic for future work. Additional revisions are needed to include the results of the karyotype analysis of other cell lines in this paper.

**Reviewer #2**

Thank you for reviewing the manuscript and interest in our study. We agree with your comment, and the manuscript was thoroughly revised based on your suggestion.

The MS “Non-viral derivation of induced pluripotent stem cells from the canine umbilical cord” describes the derivation and characterization of canine iPSCs.
The authors provide a thorough iPSC characterization and there is no doubt that iPSC generation was successful. However, one point should be clarified.
For some reason karyotype analysis was not performed. Chromosome stability (or instability) is one of the most important cell type characteristics. Veterinary usage of produced iPSC is possible only if they have normal karyotype. The authors suppose that produced iPSC line in primed. If it has XX karyotype, X-chromosome inactivation analysis would provide important clues to the pluripotency type, naïve or primed. Karyotype analysis must be performed.
I’d suggest to use proofreading services to correct English style.
Overall, the MS needs major revision, as new experimental data are necessary.

**Response:** As you suggested, karyotyping is essential for the clinical application of canine iPSCs. Following this study, experiments were conducted to confirm the reproducibility of iPSC generation. Then, karyotyping of canine umbilical cord-derived iPSCs from other individuals revealed that they contained cell lines with normal chromosome numbers. However, the cell lines in the present study have not undergone karyotyping analysis, which is a topic for future work. Additional revisions are needed to include the results of the karyotype analysis of other cell lines in this paper.

Minor points:
L51-53 “however, virus-free canine iPSCs have not yet been established… and virus-free canine iPSCs were successfully generated. [12,13].” There is a logical problem – virus-free iPSCs were not established, but at the same time they are.

**Response:** Thank you for pointing this out. The error in the text has been corrected as follows:

Several studies had previously reported on the establishment of canine iPSCs using viral vectors [10,11]; however, virus-free canine iPSCs were not established. Therefore, the clinical-grade canine iPSCs generation method using episomal vectors was established, and virus-free canine iPSCs were successfully generated in 2021 [12,13]. (line: 50-53)

L61-61 Actually skin samples could be harvested just once, that would give millions fibroblasts for many iPSC lines. Why multiple harvests?

**Response:** To apply canine iPSCs to treatment, it is necessary to prepare many iPSC lines and select cell lines that can differentiate into cells suitable for treatment. In addition, the properties of iPSCs may vary depending on the individual cell source, and it is possible that no iPSC line suitable for treatment can be obtained from a single dog. Therefore, in order to establish iPSC lines that can be used for treatment, it is necessary to harvest skin tissue frequently from multiple dogs, which is very invasive for the animal. However, canine umbilical cord tissue is waste tissue, which makes it possible to collect tissue from multiple dogs noninvasively. This is a major advantage of using umbilical cord tissue for iPSC generation.

L313 At the Discussion section shall be a paragraph with the explanation, why there were no endoderm. Is it expected? Is it normal for canine PSC teratomas? I’d expect endoderm derivatives as well as there were several teratomas, not one.

**Response:** In our experience, canine iPSCs tend to have difficulty differentiating into endoderm-derived tissues in teratoma formation. In the past, when we formed teratomas using our established canine skin-derived iPSC lines, endoderm-derived tissues were observed, but the umbilical cord-derived iPSCs in the present study did not differentiate into endoderm-derived tissues. Further investigation is needed to determine whether this is due to differences in iPSC lines or the source tissue of the iPSCs. However, the results of *in vitro* differentiation into three germ layers were shown in this study. In addition, since differentiation into two or more germ layers is recognized as a teratoma, the iPSCs established in this study were proven to have the ability to form teratomas.

L335-335. Actually, as there are MSCs in the umbilical cord as well as fibroblasts, the iPSCs were possible produced from fibroblasts. Human fibroblasts and MCSs are indistinguishable morphologically and by marker expression, I suppose it applies for canine ones too. The only way to test the cell type is adipo-, osteo- and chondrocyte differentiation.

**Response:** Recent reports have shown that fibroblasts also can differentiate into bone, cartilage, and adipocytes, making it difficult to clearly distinguish fibroblasts from MSCs even after confirming their ability for differentiation into the three cell types (Soundararajan M, Kannan S. J Cell Physiol. 2018 Dec;233(12):9099-9109.). Since MSCs may be included in the group of fibroblasts as a major framework, we decided to describe them as umbilical cord-derived fibroblasts in this study.

L386 iPSCs would be autologous to the dog from whose umbilical cord they were produced.

**Response:** Thank you for pointing this out. The error in the text has been corrected as follows:

As such, one important limitation of this study is that it is difficult to use autologous umbilical cord-derived iPSCs unless the umbilical cord were preserved at birth, and allogeneic iPSCs are used for treatment. (line: 401**-**403)

**Reviewer #3**

Thank you for reviewing the manuscript and interest in our study. We agree with your comments, and the manuscript was thoroughly revised based on your suggestions.

Major Point,
Canine iPS cells were generated from umbilical cord-derived cells. However, it is unclear whether these are from the umbilical cord of the same individual or from multiple individuals. Generating iPS cells from multiple umbilical cords is desirable to confirm the reproducibility of the experiment.

**Response:** As you suggested, it is desirable to confirm reproducibility by collecting canine umbilical cord tissue from multiple individuals and generating iPSCs. Therefore, following this study, we conducted experiments to verify the reproducibility of iPSC generation and succeeded in generating canine umbilical cord-derived iPSCs from other individual samples. In this study, we succeeded in generating the world's first canine umbilical cord-derived iPSCs, and this paper aims to show the properties of the first cell line. Thus, we added to the Discussion that experiments are needed to confirm the reproducibility (line: 391-393).

Page 16, Line 257: "Finally, four lines of canine umbilical cord-derived iPSCs were successfully generated (HOA1 #1, #2, #3, and #4), of which HOA1#3 was used for further characterization." is written, and the characteristics were analyzed from only one cell line. However, it should be investigated whether multiple cell lines meet the properties of iPS cells.

**Response:** As you pointed out, it is desirable to analyze the properties of multiple canine umbilical cord-derived iPSC lines and present all the data. However, since the purpose of this paper is to show the successful generation of iPSCs from the canine umbilical cord, we presented the data of one iPSC line that could be stably maintained and cultured. Thus, we added to the Discussion that further study is needed to confirm the characteristics of multiple iPSC lines in the future. (line: 391-393).

Page 21, Line 343: "In addition, the generation efficiency of canine umbilical cord-derived iPSCs was higher than that of dermal fibroblast-derived iPSCs." is stated, but there is insufficient data on the generation efficiency. Please provide specific data showing that umbilical cord-derived iPSCs have a higher generation efficiency than fibroblasts.

**Response:** Thank you for your suggestion. It is necessary to present data showing that the generation efficiency of umbilical cord-derived iPSCs is superior to that of skin. Compared with the generation efficiency data of canine skin-derived iPSCs previously generated in our laboratory, it is clear that the establishment efficiency of canine umbilical cord-derived iPSCs is superior to that of skin-derived iPSCs. However, since the purpose of this paper is to show the successful generation of iPSCs from the canine umbilical cord, the comparison data of the generation efficiency was not included in this study. Therefore, we added “data not shown” to this sentence (line: 350).

Page 22, Line 363: Data on SSEA-1 and SSEA-3 are shown and discussed. However, data on SSEA-4 is required.

**Response:** As you pointed out, a statement regarding the SSEA4 results is also necessary. In this study, we found that the expression of SSEA4 in canine umbilical cord-derived iPSCs is identical to previous reports of canine iPSCs. Thus, it was reconfirmed that canine iPSCs do not express SSEA4. We added relevant sentences to the Discussion (line: 368-369, 381-383).

Page 23, Line 379: The limitation states that karyotype staining was not performed. I believe that karyotype staining is necessary in this study as well.

**Response:** As you indicated, karyotyping is essential for the clinical application of canine iPSCs. Following this study, experiments were conducted to confirm the reproducibility of iPSC generation. Then, karyotyping of canine umbilical cord-derived iPSCs from other individuals revealed that they contained cell lines with normal chromosome numbers. However, the cell lines in the present study have not undergone karyotyping analysis, which is a topic for future work. Additional revisions are needed to include the results of the karyotype analysis of other cell lines in this paper.

Minor Point

Page 8, Line 114. Is the U-23 condition a device setting?

**Response:** We added in the text that U-23 is a device setting (line: 117).

Page 20, Line 331. In this study, it is stated that fibroblasts were cultured, but are these different cells from MSCs? Please consider whether to prove the cell origin.

**Response:** Recent reports have shown that fibroblasts also can differentiate into bone, cartilage, and adipocytes, making it difficult to clearly distinguish fibroblasts from MSCs even after confirming their ability for differentiation into the three cell types (Soundararajan M, Kannan S. J Cell Physiol. 2018 Dec;233(12):9099-9109.). Since MSCs may be included in the group of fibroblasts as a major framework, we decided to describe them as umbilical cord-derived fibroblasts in this study.

Fig. 6. Please explain what the asterisks indicate.

**Response:** We added the explain what the asterisks indicate (line:290-291).

Fig. 8 Was there no significant difference?

**Response:** There was no significant difference. We added the explain what the asterisks indicate (line:311-312).
