# Supplementary material for: Non-viral derivation of induced pluripotent stem cells from the canine umbilical cord
Source: PLoS One. 2025 Jun 18;20(6):e0315161. doi: 10.1371/journal.pone.0315161 (PMC12176200; doi:10.1371/journal.pone.0315161)
Supplement: Attachment — Submitted filename: Response_to_Reviewer_auresp_2.docx [file pone.0315161.s004.docx]

May 13, 2025

To Editor,

Thank you so much for your insightful review of this study. We agree with all of your comments, and the manuscript was thoroughly revised based on your suggestions. The line number in each response was shown as the number in the revised manuscript.

**Reviewer #1**

Thank you very much for your careful review of our manuscript. We have received a lot of input from you, which has been very helpful.

**Reviewer #2**

Thank you for reviewing the manuscript. The manuscript was revised based on your suggestion.

You have answered most of my questions, though I disagree with the idea that fibroblasts ans MSCs are the same cell type, I'd suggest at least to mention the articles supporting that notion in the discussion section, as the cell identity of reprogrammed cells is really important for the iPSC generation.

**Response:**

As you suggested, we added a sentence in the Discussion as follows:

In addition, it has been reported that MSCs may be included in the fibroblast group as a larger framework [16]. (line: 346-347)

The most important criticism is that you have not provided cytogenetical analysis of the generated iPSCs. Cytogenetics is one of the primary characteristics of the iPSC lines, you must provide it. It is not "a topic for future work" as in your response. And it is not just a technical issue, human and mouse pluripotent stem cells tend to accumulate chromosome anomalies due to certain mechanisms relevant to pluripotent and not somatic cells. Without cytogenetics iPSC line characterization is not complete.

**Response:**

As you pointed out, we performed a karyotype analysis and added text to the Materials and Methods, Results, and Discussion sections of the manuscript. (line: 228-231, 327-330, 400-409)

**Reviewer #3**

The author wrote, "Since the purpose of this paper is to show the successful generation of iPSCs from the canine umbilical cord, we presented the data of one iPSC line that could be stably maintained and cultured. "

If so, the karyotype of this important cell line should be investigated.

If, for some reason, the karyotype cannot be examined in this cell line, please provide the karyotype of another generated cell line.

**Response:**

As you pointed out, we performed a karyotype analysis and added text to the Materials and Methods, Results, and Discussion sections of the manuscript. (line: 228-231, 327-330, 400-409)
